# Supplementary material for: Analysis of the Whole-Exome Sequencing of Tumor and Circulating Tumor DNA in Metastatic Melanoma
Source: Cancers (Basel). 2019 Nov 29;11(12):1905. doi: 10.3390/cancers11121905 (PMC6966626; doi:10.3390/cancers11121905)
Supplement: Supplementary file 1 [file cancers-11-01905-s001.zip › cancers-620854-final-supplementary/cancers-620854-final-supplementary.docx]

Supplementary Materials

Analysis of the Whole-Exome Sequencing of Tumor and Circulating Tumor DNA in Metastatic Melanoma

Russell J. Diefenbach, Jenny H. Lee, Dario Strbenac, Jean Y. H. Yang, Alexander M. Menzies, Matteo S. Carlino, Georgina V. Long, Andrew J. Spillane, Jonathan R. Stretch, Robyn P. M. Saw, John F. Thompson, Sydney Ch’ng, Richard A. Scolyer, Richard F. Kefford and Helen Rizos





**Figure S1.** Histograms of reading depth distribution in target regions covered by WES for patient matched gDNA and ctDNA.





**Figure S2.** Pearson correlation of total plasma cfDNA extracted from melanoma patients with copy number of ctDNA determined by ddPCR.





**Figure S3.** Comparison of time between biopsies of patient matched genomic DNA and circulating tumor DNA versus concordance (% overlap of SNVs from WES).





**Figure S4.** Degree of Pearson correlation between the MAF of SNVs common to patient matched gDNA and ctDNA as identified by WES.

**Table S1–S5.** Please view at the excel file.

| 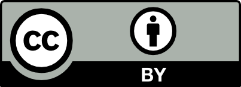 | © 2019 by the authors. Licensee MDPI, Basel, Switzerland. This article is an open access article distributed under the terms and conditions of the Creative Commons Attribution (CC BY) license (http://creativecommons.org/licenses/by/4.0/). |
| --- | --- |
